# Past Trauma Is Associated With a Higher Risk of Experiencing an Epileptic Seizure as Traumatic in Patients With Pharmacoresistant Focal Epilepsy

**DOI:** 10.3389/fneur.2021.669411

**Published:** 2021-07-08

**Authors:** Sara Mariotti, Damien Valentin, Deniz Ertan, Louis Maillard, Alexis Tarrada, Jan Chrusciel, Stéphane Sanchez, Raymund Schwan, Jean-Pierre Vignal, Louise Tyvaert, Wissam El-Hage, Coraline Hingray

**Affiliations:** ^1^Pôle Hospitalo-Universitaire de Psychiatrie d'Adultes du Grand Nancy, Centre Psychothérapique de Nancy, Laxou, France; ^2^Université de Lorraine, Faculté de Médecine, Vandœuvre-lès-Nancy, France; ^3^Université de Lorraine, CNRS, CRAN, UMR 7039, Nancy, France; ^4^Etablissement la Teppe Tain l'Hermitage, Tain-l'Hermitage, France; ^5^CHRU de Nancy, Département de Neurologie, Nancy, France; ^6^Pôle Information Médicale Évaluation Performance, CH de Troyes, Troyes, France; ^7^INSERM U1114, Université de Strasbourg, Strasbourg, France; ^8^UMR 1253, iBrain, Université de Tours, INSERM, Tours, France; ^9^CHU de Tours, Tours, France

**Keywords:** drug-resistant focal epilepsy, trauma, traumatic experienced seizure, psychiatric comorbidities, postepileptic seizure posttraumatic stress disorder, posttraumatic stress disorder

## Abstract

**Objective:** The present study aimed to evaluate the prevalence of traumatic experienced seizures (TES) and of postepileptic seizure PTSD (PS-PTSD) in patients with pharmacoresistant focal epilepsy and to explore the determining factors of TES.

**Methods:** We conducted an observational study enrolling 107 adult refractory epilepsy patients. We used the DSM-5 criteria of traumatic events and PTSD to define TES and PS-PTSD. We assessed all traumatic life events unrelated to epilepsy, general and specific psychiatric comorbidities, and quality of life.

**Results:** Nearly half (*n* = 48) of the 107 participants reported at least one TES (44.85%). Among these, one-third (*n* = 16) developed PS-PTSD. The TES group was more likely to experience traumatic events unrelated to epilepsy (*p* < 0.001), to have generalized anxiety disorder (*p* = 0.019), and to have specific psychiatric comorbidities [e.g., interictal dysphoric disorder (*p* = 0.024) or anticipatory anxiety of seizures (*p* = 0.005)]. They reported a severe impact of epilepsy on their life (*p* = 0.01). The determining factors of TES according to the multifactorial model were the experience of trauma (*p* = 0.008), a history of at least one psychiatric disorder (*p* = 0.03), and a strong tendency toward dissociation (*p* = 0.03).

**Significance:** Epileptic seizures may be a traumatic experience in some patients who suffer from pharmacoresistant epilepsy and may be the source of the development of PS-PTSD. Previous trauma unrelated to epilepsy and psychiatric history are determining factors of TES. These clinical entities should be explored systematically.

## Introduction

Epilepsy is defined as a chronic brain disorder characterized by an enduring predisposition to generate epileptic seizures and by the neurobiological, cognitive, psychological, and social consequences of this condition (1). Epilepsy is present in ~1% of the population, accounting for a total of 70 million people worldwide, approximately one-third of whom have refractory epilepsy (2). Psychiatric disorders have been identified in 25–50% of patients with epilepsy, with a higher prevalence among patients with poorly controlled seizures (3).

The association between epilepsy and negative life events is multidirectional and complex. Early-life stress might promote epileptogenesis during brain development with a vulnerability to limbic epilepsy (4, 5). People with posttraumatic stress disorder (PTSD) have a higher risk of developing epilepsy in the future (6). Moreover, self-reported stress is the most common seizure precipitant (7). Therefore, acute stress due to traumatic events could trigger an epileptic seizure (8). People who live in war zones (9) or in disaster-prone countries (10) are more likely to experience a seizure. PTSD is a mental health condition that is known to affect people who have experienced or witnessed a traumatic event (11). Illnesses can also be forms of trauma. Several studies have proven that acute diseases could be considered traumatic for patients, such as acute coronary syndrome (12), stroke (13, 14), asthma (15), or first-episode psychosis (16).

Two studies have evaluated whether an epileptic seizure could be perceived as a traumatic event and reported different findings. Chung and Allen (17) investigated the incidence of PTSD following epileptic seizure and called it postepileptic seizure PTSD (PS-PTSD). Their results indicated that 51% of 71 patients with all types of epilepsy met the diagnostic criteria for full-PTSD in reference to their “most traumatic seizure” according to the Posttraumatic Diagnostic Scale (PDS-5). Labudda et al. (18) used a modified version of the PDS-5 and conducted interviews to assess patients who fulfilled the criteria for PS-PTSD and asked about their worst seizure. Only 5% of the 120 patients in the sample fulfilled all criteria for PTSD.

Patients with pharmacoresistant focal epilepsy (PRFE) have more psychiatric comorbidities compared to patients with controlled epilepsy, and these associated psychiatric factors cause poorer life quality (19). Therefore, focusing on these comorbid factors, especially on traumatic dimension, could be an important resource to take actions to improve the life quality of the patients with PRFE for whom the antiepileptic treatment is limited.

Our study aimed to measure the prevalence of traumatic experience of an epileptic seizure (TES) and of PS-PTSD in patients with focal refractory epilepsy and to explore the determining factors (epileptic and psychiatric) linked to TES.

## Materials and Methods

This prospective study was conducted between November 2018 and February 2020 in the Epileptology Department of our University Hospital. We enrolled consecutive adult patients hospitalized for presurgical work-up with a confirmed diagnosis of pharmacoresistant focal epilepsy (according to the ILAE) few months before possible intracranial exploration. All patients provided written consent. We collected data based on our clinical systematic evaluation. Sociodemographic data were collected, including age, sex, marital status, education, and employment status.

### Seizure Data

We identified the age at epilepsy onset and the type of seizures (focal and/or focal to bilateral tonic–clonic seizures). Localization and lateralization of seizure foci were based on the recorded seizures during long-term video-EEG monitoring and images in all patients and additional video-SEEG (stereo-electroencephalography) for some patients. MRIs for epileptogenic lesions were sought. Data assessing ongoing antiepileptic treatment were collected. The impact of epilepsy on life and quality of life was evaluated by the Quality of Life in Epilepsy Inventory (QOLIE-31) (20), composed of seven multi-item subscales evaluating emotional well-being, social function, energy, cognitive function, seizure worry, medication effects, and overall quality of life, and by a question in which the impact of epilepsy on daily life was evaluated by the patients as absent, mildly, moderately, and severely. The most impacted life areas were also investigated, e.g., family, sentimental, working, and leisure.

### Trauma Data

#### Traumatic Experienced Seizure (TES)

The risk or fear of death or serious injury during a seizure was examined based on the definition of trauma provided by the DSM-5 (21). If this risk or fear was present, we confirmed TES and further questions were asked, such as the number of traumatic seizures experienced and which seizure was the most traumatic one (e.g., the first one, the last one, and the most serious one in terms of severity of the circumstances or the consequences). The temporal relationship between the onset of epilepsy and the first traumatic seizure was also explored.

#### Postepileptic Seizure Posttraumatic Stress Disorder

The patients who experienced TES constituted the TES group, while all other patients composed the non-TES group. In the TES group, the PCL-5 (PTSD Checklist Scale for DSM-5) (22) was used to evaluate the severity of symptoms associated with this seizure as a traumatic event. The PCL-5 is a 20-item questionnaire corresponding to the DSM-5 symptom criteria for PTSD. The self-report rating scale is 0–4 for each symptom: “Not at all,” “A little bit,” “Moderately,” “Quite a bit,” and “Extremely.” It combines four subcategories intrusion (item B, questions 1–5) avoidance behavior (item C), cognition and mood alteration (item D), and hypervigilance (item E). A provisional PTSD diagnosis can be made by treating each item rated as 2 = “Moderately” or higher as a symptom endorsed, then following the *DSM-5* (23) diagnostic rule that requires at least one B item (questions 1–5), one C item (questions 6–7), two D items (questions 8–14), and two E items (questions 15–20). Following this method, we identified a positive provisional PTSD group and a negative provisional PTSD group.

#### History of Trauma, Independent of a Seizure

The experience of traumas other than epilepsy was evaluated using the Traumatic Life Event Questionnaire (TLEQ) (24). The frequency and type of trauma and the age of occurrence were evaluated. Traumas during childhood were assessed using the TLEQ and the Childhood Trauma Questionnaire (CTQ) (23). These scales enabled the establishment of three types of trauma: sexual, physical, and psychological trauma, as well as the occurrence, the age at the first experience, and the time between the first traumatic event and the appearance of epilepsy. The PTSD part of Mini International Neuropsychiatric Interview (MINI) was used to diagnose an actual PTSD. We also investigated a past PTSD, which refers to a total remission of the PTSD symptoms at least for 1 month.

#### Dissociation

Dissociation was assessed through the Dissociative Experiences Scale (DES) (25), a 28-item self-report tool that rates the severity and frequency of dissociative experiences, which explores three subcategories of dissociative symptoms: depersonalization, amnesia, and absorption.

### Psychiatric Assessment

#### Non-specific Psychiatric Disorders

Psychiatric comorbidities were assessed through the semi-structured interview MINI (26). Depression and generalized anxiety disorder (GAD) were evaluated by two specific validated scales for patients with epilepsy: the Neurological Disorders Depression Inventory for Epilepsy (NDDIE) (27) and the GAD-7 (28), respectively. Data about ongoing psychotropic treatment were collected, as were comorbidities induced by these treatments.

#### Specific Interictal Psychiatric Disorders Associated With Epilepsy—With No Temporal Link to Seizures

We evaluated *interictal dysphoric disorder* according to Blumer's criteria, defined as the occurrence of at least three episodes lasting from a few hours to a few days, grouped together at least three of the following eight criteria: depressed mood, asthenia, atypical pain, insomnia, fear and anxiety, irritability, euphoric mood, and instability of mood (29). Anticipatory anxiety of a seizure, defined as the fear of having an epileptic seizure, was assessed. We also explored avoidance behavior linked to the fear of seizures.

#### Peri-Ictal Disorders—With a Temporal Link to Seizures

For the three major dimensions of psychiatric comorbidities (depression, anxiety, and psychosis), we assessed the presence of preictal disorder, ictal disorder, and postictal disorder.

#### Psychological Dimensions

Alexithymia was assessed through the use of the Toronto Alexithymia Scale (TAS-20) (30), which is a self-report tool with three subscales: feelings' description and identification difficulties and thoughts turned to the outside.

### Statistical Analyses

To evaluate differences between participants who experienced or did not experience TES, the Chi-square test and Fisher's exact test were used to analyze categorical variables. We used Student's *t*-test for normally distributed continuous variables with enough patients (>30) and the Mann–Whitney *U*-test for other continuous variables. Missing data were excluded from the respective analyses. A two-tailed *p*-value of < 0.05 was considered statistically significant. Since these analyses were exploratory, the Bonferroni correction was not necessary (31, 32).

We also performed a multivariate logistic regression analysis, which models the probability of a TES. The variables included were the duration of epilepsy, the existence of interictal anticipatory anxiety, previous trauma, a history of at least one psychiatric disorder, current anxious disorder, and the total DES score. Missing values were processed by multiple account assignment (MAA).

## Results

### Study Population

**Figure 1:**
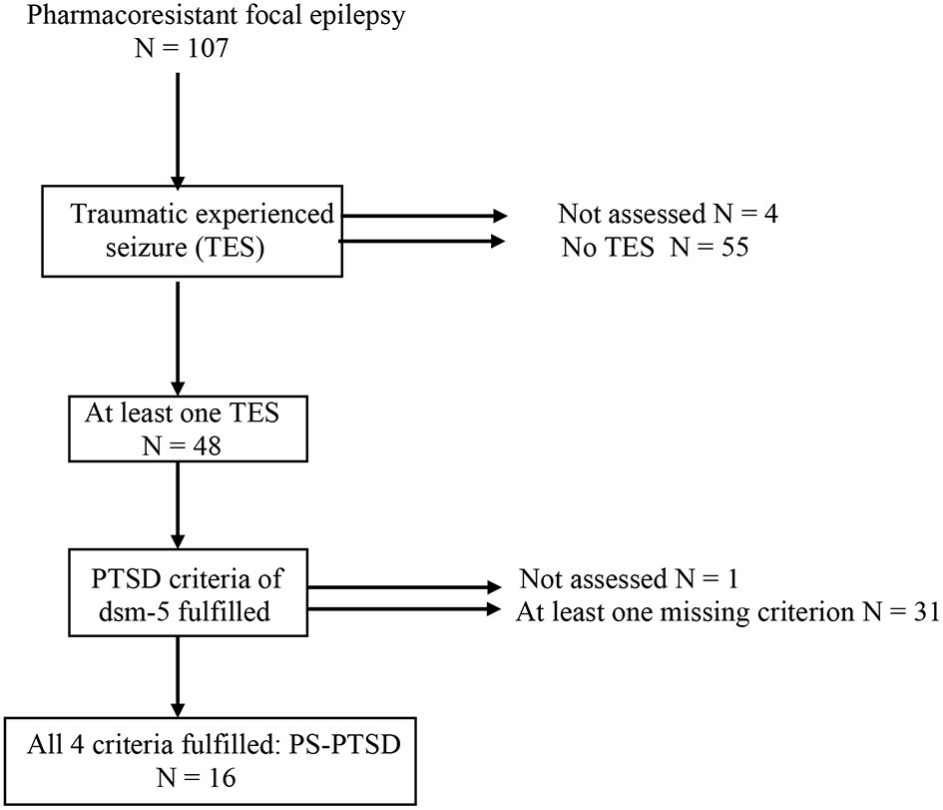
Representation of patients with/without TES and with/without PS-PTSD.

Assessments were completed by 107 patients (age 33.2 years, 18–66): 48 had a TES and 16 had PS-PTSD (Figure 1). The female-to-male ratio was 1.14. The mean age at onset of epilepsy was 17.55 years, with an average duration of evolution of 15.6 years. A majority of participants (55.14%) reported at least one traumatic experience unrelated to epilepsy in their lifetime. In 27.1% of the cases, this trauma preceded the onset of epilepsy. A majority of patients (59.81%) had an ongoing psychiatric disorder. Only 16.82% of our patients reported having mood disorders at the time of evaluation, whereas 31.77% of our population reported current anxiety disorders. Thirty-eight patients suffered from anticipatory anxiety of seizures, while 41.1% avoided at least one situation in their daily life (Supplementary Table 1).

Figure 1 presents the repartition of patients.

### Traumatic Experience of Seizures

Of the 107 participants, 48 (44.85%) experienced at least one TES, for an average of 2.79 TESs in a lifetime. Among these 48 patients, 29.16% assessed their first seizure as a TES and 41.66% considered their most serious seizure (in term of circumstances or consequence) as a TES. The average duration between the onset of epilepsy and a TES was 5.29 years (Supplementary Table 1). Of these 48 patients, 16 met all 4 criteria of PTSD diagnosis and were constituted PS-PTSD group.

#### Profile of Patients in the TES Group? Comparative Univariate Analyses

##### Demographic Data

There was no difference between the two groups in sociodemographic or educational data.

##### Characteristics of Epilepsy

The mean age at epilepsy onset as well as the frequency, lateralization, and localization of seizures did not differ significantly between the two groups. However, TES patients tended to have more frequent focal to bilateral tonic–clonic seizures (*p* = 0.06) and more frequent falls (*p* = 0.08). We observed a longer duration of epilepsy among patients without TES (17.98 vs. 11.72, *p* = 0.002). Patients without TES were more likely to experience head injury (27.27 vs. 10.41%, *p* = 0.03).

##### Impact of Epilepsy on Quality of Life

Patients in the TES group reported more severe impact of epilepsy than other patients (32.25 vs. 9.3%, *p* = 0.01). The various aspects of daily life were equally impacted between groups, except for leisure, which was more disrupted in the TES group (*p* = 0.013). On the QOLIE 31, the social function was lower in the TES group (*p* = 0.012). The TES group had overall poorer quality of life, but not significant on the following dimensions: worry about seizures (*p* = 0.055), side effects from epileptic illness, and side effects from treatments (*p* = 0.069). Data concerning patients profile are presented in Table 1.

**Table 1:** Demographics and epileptic features of patients with/without traumatic experienced seizure (TES).

	**At least one traumatic experienced seizure (*N* = 48)**	**No traumatic experienced seizure (*N* = 55)**	***p***
**DEMOGRAPHIC FACTORS**			
Sex ratio M/F, *n*	0.92	0.77	0.66^*^
Age at enrollment, years (SD)	31.45 (10.25)	34.43 (13.01)	0.20^****^
Learning disabilities, *n* (%)	13 (27.08)	16 (29.09)	0.82^*^
Marital status (single), *n* (%)	26 (54.16)	27 (49.09)	0.6^*^
Education level since primary school, years (SD)	12.87 (2.54)	12.2 (2.71)	0.15^***^
Professional activity, *n* (%)	18 (37.5)	25 (45.45)	0.41^*^
**EPILEPTIC FEATURES**			
**History**			
Neurological history—head injury, *n* (%)	5 (10.41)	15 (27.27)	0.03^*^
Age at the onset of epilepsy, years (SD)	19.54 (11.52)	16.2 (12.01)	0.15^****^
Duration of epilepsy, years (SD)	11.72 (8.81)	17.98 (11.30)	0.002^****^
**Type, frequency and localization**			
Focal seizure (focal), *n* (%)	43 (89.58)	51 (92.72)	0.73^**^
Focal to bilateral tonic–clonic seizures, *n* (%)	28 (58.33)	22 (40)	0.06^*^
Focal seizures frequency per month, *n* (SD)	44.57 (139.7)	13.47 (20.92)	0.47^***^
Focal to bilateral tonic–clonic seizures—per month, *n* (SD)	8.28 (22.08)	0.78 (1.02)	0.28^***^
Left-sided focus of epilepsy, *n* (%)	24 (57.14)	27 (52.94)	0.68^*^
Right-sided focus of epilepsy, *n* (%)	16 (38.09)	19 (37.25)	0.93^*^
Bilateral focus of epilepsy, *n* (%)	2 (4.76)	5 (9.8)	0.45^**^
Temporal lobe epilepsy, *n* (%)	30 (71.42)	39 (75)	0.69^*^
Frontal lobe epilepsy, *n* (%)	7 (16.66)	11 (21.15)	0.58^*^
Insular epilepsy, *n* (%)	3 (7.14)	7 (13.46)	0.5^**^
Posterior lobe epilepsy, *n* (%)	5 (11.9)	9 (17.3)	0.46^*^
**Severity**			
Loss of consciousness during a seizure, *n* (%)	24 (50)	35 (64.81)	0.13^*^
Urine or feces loss during a seizure, *n* (%)	10 (20.83)	10 (18.51)	0.76^*^
Fall during a seizure, *n* (%)	24 (50)	18 (33.33)	0.08^*^
Injuries during a seizure, *n* (%)	20 (41.66)	20 (37.03)	0.63^*^
**IMPACT OF EPILEPSY ON LIFE**			
**Subjective impact**			
Impact on daily life evaluated as severe, *n* (%)	10 (32.25)	4 (9.30)	0.01^*^
Family life affected by epilepsy, *n* (%)	7 (22.58)	5 (11.62)	0.20^*^
Sentimental life affected by epilepsy, *n* (%)	6 (19.35)	7 (16.27)	0.73^*^
Working life affected by epilepsy, *n* (%)	24 (77.41)	30 (69.76)	0.46^*^
Leisure affected by epilepsy, *n* (%)	22 (70.96)	18 (41.86)	0.013^*^
**Quality of life (QOLIE 31)**			
Worry about seizures, score (SD)	42.01 (28.41)	52.24 (25.01)	0.055^***^
General quality of life, score (SD)	51.36 (20.25)	57.34 (19.64)	0.16^***^
Emotional wellness, score (SD)	60.09 (23.03)	66.23 (21.65)	0.22^***^
Vitality and energy feeling, score (SD)	46.66 (17.91)	47.49 (19.91)	0.57^***^
Memory, cognitive disorders, score (SD)	40.965 (19.85)	46.89 (25.35)	0.12^***^
Side effects from treatments, score (SD)	44.92 (24.74)	53.45 (26.50)	0.069^***^
Social functioning, score (SD)	51.08 (25.65)	63.13 (23.38)	0.012^***^

* *Chi 2*;

** *Fisher exact test*;

*** *Mann–Whitney U-test*;

**** *Student's t-test*.

*SD, standard deviation; QOLIE, quality of life in epilepsy inventory*.

##### Psychiatric Disorders

Patients in the TES group were more likely to have psychiatric disorders than non-TES patients (68.75 vs. 52.72%, respectively, *p* = 0.003) and used to take more psychotropic drugs than other patients (*p* = 0.04).

*Mood Disorders*. According to the MINI or the NDDIE, there was no difference between the two groups in terms of general mood disorders. Regarding specific mood disorders linked with epilepsy, interictal dysphoric disorders were more often found among TES patients (33.33 vs. 14.54%, *p* = 0.024) as well as preictal mood disorders (14.58 vs. 0%, *p* = 0.003).

*Anxiety Disorders*. Current anxiety disorders were more frequent in patients in the TES group (47.91 vs. 20%, *p* = 0.002). Specifically, it concerned current GAD (20.83 vs. 5.45%, *p* = 0.019) according to MINI, but the two groups did not differ in terms of GAD according to a positive score on the GAD-7 scale (*p* = 0.08). People in the TES group were more likely to present anxious disorders related to epilepsy, such as anticipatory anxiety (70 vs. 37.2%, *p* = 0.005), ictal anxiety—twice as likely to occur in patients in the TES group (*p* = 0.009)—and avoidance behaviors related to anxiety (*p* = 0.009). See Table 2.

**Table 2:** Psychiatric features of patients with/without traumatic experienced seizure (TES).

	**At least one traumatic seizure (*N* = 48)**	**No traumatic seizure (*N* = 55)**	***p***
**Personal psychiatric history**			
Psychiatric follow-up, *n* (%)	22 (45.83)	23 (41.81)	0.68^*^
Hospitalization for psychiatric care, *n* (%)	8 (16.66)	4 (7.27)	0.13^*^
Personal history of suicide attempt, *n* (%)	7 (14.58)	5 (9.09)	0.38^*^
Number of past psychiatric comorbidities, mean (SD)	1.58 (1.11)	0.87 (1.07)	<0.001^***^
**Current psychiatric comorbidities**			
At least one current pathology (MINI), *n* (%)	33 (68.75)	29 (52.72)	0.09^*^
Number of current psychiatric comorbidities, mean (SD)	1 (0.93)	0.74 (0.83)	0.12^***^
Number of Current psychotropic treatment, mean (SD)	0.375 (0.78)	0.09 (0.28)	0.04^***^
Antidepressants, *n* (%)	7 (14.58)	3 (5.45)	0.18^**^
Antipsychotics, *n* (%)	5 (10.41)	1 (1.81)	0.09^**^
Anxiolytics, *n* (%)	6 (12.5)	1 (1.81)	0.04^**^
**Mood disorders**			
Current depressive disorder (MINI), *n* (%)	8 (16.66)	4 (7.27)	0.13^*^
Depression according NDDIE score ≥16, *n* (%)	11 (26.19)	6 (11.76)	0.07^*^
**Specific mood disorder linked with epilepsy**			
Interictal dysphoric disorder, *n* (%)	16 (33.33)	8 (14.54)	0.024^*^
Pre-ictal mood disorder, *n* (%)	7 (14.58)	0 (0)	0.003^**^
Ictal mood disorder, *n* (%)	3 (6.25)	0 (0)	0.09^**^
Post-ictal mood disorder, *n* (%)	10 (20.83)	6 (10.9)	0.16^*^
**Anxiety disorder**			
At least one current anxiety disorder (MINI), *n* (%)	23 (47.91)	11 (20.0)	0.002^*^
Panic disorder, *n* (%)	3 (6.25)	5 (9.09)	0.72^**^
Agoraphobia, *n* (%)	7 (14.58)	2 (3.63)	0.07^**^
Generalized anxiety disorder, *n* (%)	10 (20.83)	3 (5.45)	0.019^*^
Social phobia, *n* (%)	6 (12.5)	2 (3.63)	0.14^**^
GAD according GAD-7 score ≥8, *n* (%)	14 (33.33)	9 (17.64)	0.08^*^
**Specific anxiety disorders linked with epilepsy**			
Pre-ictal anxiety, *n* (%)	21 (43.75)	14 (25.45)	0.050^*^
Interictal/anticipatory anxiety of a seizure, *n* (%)	21 (70)	16 (37.2)	0.005^*^
Ictal anxiety, *n* (%)	22 (45.83)	12 (21.81)	0.009^*^
Post-ictal anxiety, *n* (%)	10 (20.83)	5 (9.09)	0.09^*^
Behavior of limitation or avoidance, *n* (%)	26 (54.16)	16 (29.09)	0.009^*^
**Obsessive-Compulsive**			
Obsessive-compulsive disorder, *n* (%)	3 (6.25)	1 (1.81)	0.33^**^
**Psychotic disorders**			
Psychotic disorder (MINI), *n* (%)	4 (8.33)	6 (10.90)	0.74^**^
Post-ictal psychosis, lifetime, *n* (%)	1 (2.08)	0 (0)	0.46^**^
**Addictive disorders**			
Alcohol addiction (MINI), *n* (%)	0 (0)	3 (5.45)	0.24^**^
Cannabis addiction (MINI), *n* (%)	2 (4.16)	3 (5.45)	1^**^
**Alexithymia**			
Alexithymia (according TAS score >61), *n* (%)	14 (33.33)	2 (4.76)	<0.001^*^
Feelings' description difficulties mean score (SD)	13.97 (4.36)	12.11 (3.45)	0.035^****^
Feelings' identification difficulties mean score (SD)	18.8 (6.93)	16 (5.23)	0.041^****^
Thoughts turned to the outside mean score (SD)	20.59 (4.46)	20.47 (3.49)	0.89^****^

* *Chi 2*;

** *Fisher exact test*;

*** *Mann–Whitney U-test*;

**** *Student's t-test*.

*SD, standard deviation; GAD, generalized anxiety disorder; NDDI-E, neurological disorders depression inventory for epilepsy; TAS, Toronto alexithymia scale; MINI, mini international neuropsychiatric interview*.

##### Addictive and Psychotic Disorders

No significant differences were found.

##### Alexithymia

Patients in the TES group were more likely to develop alexithymia: 33.33 vs. 4.76%, respectively (*p* < 0.001).

##### History of Trauma

The majority (81.25%) of the TES group experienced a previous trauma (vs. 47.27% in the non-TES group, *p* < 0.001), with a higher average number of traumatic experiences (1.7 vs. 0.7, *p* < 0.001) and a higher proportion of patients who experienced several traumatic events (52.08% vs. 25.45%, *p* = 0.005). The TES group was more likely to experience trauma before the onset of epilepsy (37.5 vs. 20%, *p* = 0.048) and to have a history of PTSD unrelated to epilepsy (20.83 vs. 7.27%, *p* = 0.045)

Thirty-eight patients in the TES group (79.16%) presented at least one PTSD symptoms related to a TES, and 16 patients had the four criteria. The average total score for dissociation was significantly higher among patients in the TES group (*p* < 0.001). See Table 3.

**Table 3:** Traumatic characteristics of patients with/without traumatic experienced seizure (TES).

	**At least one traumatic seizure (*N* = 48)**	**No traumatic seizure (*N* = 55)**	***p***
**Trauma data**			
Previous trauma, *n* (%)	39 (81.25)	26 (47.27)	<0.001^*^
Number of previous trauma, mean (SD)	1.70 (1.41)	0.70 (0.88)	<0.001^***^
Age at first trauma, years (SD)	17.37 (10.81)	15.22 (9.64)	0.71^***^
Onset of trauma before epilepsy, *n* (%)	18 (37.5)	11 (20)	0.048^*^
One trauma, *n* (%)	14 (29.16)	12 (21.81)	0.39^*^
Several trauma, *n* (%)	25 (52.08)	14 (25.45)	0.005^*^
Trauma during adulthood			
Sexual, *n* (%)	0 (0)	1 (1.81)	0.34^**^
Physical, *n* (%)	8 (16.66)	3 (5.45)	0.06^*^
Psychological/emotional, *n* (%)	19 (39.58)	6 (10.9)	<0.001^*^
Trauma during childhood			
Sexual, *n* (%)	7 (14.58)	5 (9.09)	0.38^*^
Physical, *n* (%)	14 (29.16)	7 (12.72)	0.038^*^
Psychological/emotional, *n* (%)	23 (47.91)	16 (29.09)	0.049^*^
**PTSD unrelated to epilepsy**			
Past PTSD, *n* (%)	10 (20.83)	4 (7.27)	0.045^*^
Actual PTSD, *n* (%)	4 (8.33)	1 (1.81)	0.18^**^
**Post Seizure PTSD (PS-PTSD)**			
At least one criteria significant of PS-PTSD (PCL 5) n (%)	38 (79.16)	-	**-**
All 4 criteria fulfilled: PS-PTSD n (%)	16 (33.33)	-	**-**
Total mean score of PCL-5 (SD)	18.44 (15.30)	-	**-**
**Dissociation tendency(DES)**			
Total mean score (SD)	13.29 (8.06)	7.18 (7.70)	<0.001^***^
Depersonalization, mean score (SD)	13.84 (9.38)	7.02 (7.38)	0.001^***^
Amnesia, mean score (SD)	9.15 (7.97)	4.98 (5.88)	0.01^***^
Absorption, mean score (SD)	20.13 (13.88)	10.58 (11.68)	0.002^***^

* *Chi 2*;

** *Fisher exact test*;

*** *Mann–Whitney U-test*;

**** *Student's t-test*.

*SD, standard deviation; PTSD, post-traumatic stress disorder; DES, dissociative experiences scale; PCL-5, post-traumatic stress disorder checklist for DSM-5*.

*Bold values correspond to significant results*.

#### Determining Factors of TES? A Multivariate Analysis

Three factors appeared as determining factors of TES: the existence of a previous trauma (*p* = 0.008), a history of at least one psychiatric disorder (*p* = 0.03), and a high total score on the DES (*p* = 0.03) (Table 4).

**Table 4:** Multivariate logistic regression analysis modeling the probability of a traumatic experienced seizure (TES).

	**Multivariate (CI 95%) OR^a^**	**Multivariate *p*-value^a^**
Duration of epilepsy, years^b^	0.944 (0.886–1.006)	0.08
Interictal anticipatory anxiety of a seizure	3.111 (0.687–14.083)	0.14
Existence of previous trauma	4.823 (1.53–15.211)	**0.008**
At least one history of psychiatric disorder	5.565 (1.178–26.285)	**0.03**
At least one current anxious disorder	1.171 (0.328–4.183)	0.81
Dissociation, DES total score^b^	1.116 (1.014–1.226)	**0.03**

a *Missing values processed by Multiple Account Assignment (MAA)*.

b *Coefficient for additional consultation (continuous variable)*.

*DES, dissociative experiences scale; OR, odds ratio; CI, confidence interval*.

*Bold values correspond to significant results*.

### Postepileptic Seizure Posttraumatic Stress Disorder

One-third (*n* = 16) of patients who experienced a TES developed a provisional PS-PTSD, which represented 14.95% of our total population. Due to the small number of patients, we did not perform comparative or multivariate analyses.

## Discussion

Half of the 107 patients had at least one TES, and one-third of patients in the TES group developed a potential/provisional PS-PTSD, which was 14.95% of our total population PRFE. Two previous studies have shown that an epileptic seizure could be experienced as traumatic. Labudda et al. (18) found that only 5% of included patients had PTSD in a population of difficult-to-treat individuals who had not been treated by surgery. In contrast, Chung and Allen (17) concluded that 51% of the 71 patients with all types of epilepsy met PTSD criteria. We found intermediary results. These differences between studies might be due to the methodological differences (such as assessment of PTSD). In our study, we did an exhaustive psychiatric interview by using event checklist to assess if patients experienced one or more traumatic events; then, the four core-symptoms of PTSD (avoidance, negative changes in mood and cognition, reexperience, and hyper-arousal) were sought by interview, which allowed us to justify PTSD diagnosis, according to DSM-V criteria. Moreover, patients who reported symptoms were assessed through the use of PCL-5 in order to rate their severity. Chung et al. (33) did the PTSD diagnosis based on PDS DSM-3 version. The PTSD diagnosis is therefore only based on a self-reported questionnaire without a standardized psychiatric interview. Moreover, the authors asked the participants to complete PDS regarding their most traumatic seizure without checking if this event represent a traumatic experience based on DSM criteria. Therefore, we believed that, there might be an overvaluation or the prevalence of the patients with post-seizure PTSD. Moreover, we believe that there is also an overvaluation for the control group with 24% of participants with PTSD. Regarding Labudda et al. (18), the authors also used the PDS self-questionnaire. The authors asked the participants to remember their worst seizure and if they could identify one or more extraordinarily upsetting seizure. If the patients could identify a distressing seizure, then they checked if this event represented a traumatic experience according to DSM-4. We believe that their methodological approach is *a priori* much more restrictive. We also believe that differences in the PS-PTSD rates among the three studies arose from the fact that populations were not comparable: our study included specifically patients with pharmacoresistant focal epilepsy. By contrast, the two other studies included patients with epilepsy, whether it concerned drug-resistant epilepsy or not.

### Trauma History

Additional studies suggested that there are multidirectional links between trauma and epilepsy. The patients with epilepsy who perceived stress as a trigger for seizures were more likely to have a history of childhood maltreatment (34, 35). In a study of pharmacoresistant epilepsy patients, 75% reported having experienced a traumatic event other than a seizure, and 20% reported that their first seizure arrived in a traumatic context and showed more PTSD symptoms (33). In our study, we showed that patients with TES had more past trauma.

As a result of a traumatic history, TES patients reported more regular dissociative experiences in daily life. We assumed that the patients who have higher dissociative tendencies in their everyday life have already experienced regularly some altered forms of consciousness, automatic behaviors, etc. Therefore, perhaps, for these patients, experiencing a loss of consciousness during an epileptic seizure is more traumatic. We suppose that these patients who have higher level of dissociation might experience more traumatic seizures compared to patients with lower level of dissociation.

On a neurobiological perspective, the hypothalamo–pituitary–adrenal (HPA) axis is activated in stressful situations, followed by the release of corticosteroid hormones. Increased activity in the HPA axis has been observed in early life traumas (36). Chronic or repeated stress could exacerbate epileptogenesis, inducing a greater predisposition for further stress experiences to trigger seizures (37). People who underwent maltreatment during childhood have a reduction of the hippocampus volume by ~6% (38, 39). A smaller hippocampus could result in less efficient mnesic integration of the traumatic event in its chronology, leading to pathological intrusions. Amygdala overactivation induces fear and hypervigilance symptoms of PTSD. Therefore, a previous trauma alters these functions and could weaken a patient who may perceive a second unpredictable event, such as an epileptic seizure, as a new trauma.

### Psychiatric Comorbidities

Suffering from epilepsy and anxious disorder might increase the risk of subjectively experiencing an epileptic seizure as more traumatic as a result of a catastrophic scenario made by anticipation or after a seizure. TES could be a consequence of this anxiety, or even of depression.

Moreover, the link between psychiatric comorbidities and epilepsy could be a consequence of TES. Therefore, experienced TES might increase the risk of developing a psychiatric pathology, especially if this experience induces social isolation. These psychiatric comorbidities might also be a confounding factor with trauma history, which is known to ensure these psychiatric comorbidities even before seizure appearance.

Our study showed that half of the patients who experienced a traumatic seizure had anticipatory anxiety about a seizure, a subjective symptom described as a day-to-day persistent fear, dread, or worries to have a seizure. A previous study demonstrated that this anticipatory anxiety was not correlated with the objective severity, frequency, or localization of seizures but was related to trauma history (40). A traumatic experience could induce more anticipatory anxiety, avoidance behavior, and ictal anxiety among patients with TES. We found that the TES group has experienced higher rates of peri-ictal psychiatric symptoms compared to the non-TES group. Possibly, having a TES could increase the seizure related anxiety and consequently the peri-ictal complications. However, we do not have a chronological data and therefore we could not demonstrate any causal links. Alternatively, general mood and anxiety disorders that were more frequent in TES patients could increase the likelihood of peri-ictal symptoms (41).

Chung and Allen (17) demonstrated that alexithymia was more common among patients with TES. Similarly, our results showed that one-third of TES patients had a diagnosis of alexithymia. Alexithymia might constitute a defense mechanism for these people to protect themselves from anxiety, fear, and negative emotions that a seizure could generate. Alexithymia was also associated with higher rates of affective disorders (42). Bewley et al. (41) suggested that alexithymia could be due to neurologic deficits induced by epilepsy, such as right cortical lesions, dysfunction of the right cerebral hemisphere, or frontal lobe dysfunction (43). Alexithymia was also described in link with trauma: perhaps people who have difficulties verbalizing their emotions could be more vulnerable in front of a danger or another perturbating situation.

### Quality of Life

In our study, TES patients had a significantly lower score on the social functioning dimension of the QOLIE-31 and seemed more concerned about their seizures, which could explain the higher level of anticipatory anxiety. A decrease in social interactions might be the consequence of the traumatic experience of seizures, or that altered social interactions involve social isolation due to psychiatric disease (44). Alternatively, this social alteration could also be induced by trauma. Trauma creates a feeling of unsafety. Furthermore, TES patients have slightly more tendency to suffer from the side effects of antiepileptic drugs (45, 46).

Our results showed that TES was not significantly correlated with the type of seizure frequency, severity, or localization. However, we found that TES patients tended to have more frequent focal to bilateral tonic–clonic seizures and presented a higher incidence of fall during seizure. These could increase the severity of the seizures and therefore could be considered as important contributing factors for TES.

Moreover, there was a link between TES and duration of the disease. The time since onset of epilepsy was shorter in the TES group than in the non-TES group. Possibly, there was a memorization bias because TES was closer in time, so patients might remember them more precisely. It could also reflect a habituation phenomenon; people with a longer history of epilepsy would be more psychologically prepared for a seizure that is experienced as less traumatic than those whose onset is more recent. Alternatively, patients in the TES group might have an earlier consultation for their seizure because of their traumatic nature. An earlier management of the seizures might also explain this onset difference.

Furthermore, most of the patients examined herein had temporal epilepsy, which is related to the hippocampus. In some forms of focal epilepsy, as in temporal epilepsy, hippocampal sclerosis is present, which could also induce symptoms of trauma and PTSD by the mechanism that we described above. Further studies focusing on structural hippocampal and amygdala abnormalities in patients with TES could contribute to our current knowledge on TES pathophysiology.

This study has some limitations, such as a low number of included patients (107) and a small number for multidimensional analysis of PS-PTSD. Further studies are necessary to confirm these links in larger samples. Moreover, our study sample included only the patients with PRFE. Therefore, our findings are not representative of people with general idiopathic or drug responsive epilepsy. Our results must be interpreted with caution. The participants may have been affected by memorization bias. There could be some confounding factors between a TES vulnerability predisposition and its consequences. Conscious awareness during seizures is an important factor for traumatically experienced seizures. The memory of seizure might influence the stress related to it. In the current study, we did not evaluate the level of consciousness. Further studies should investigate the patient's consciousness during seizure. Moreover, we did not investigate precisely the seizure type (focal or focal to bilateral tonic–clonic seizures) causing TES among our patients.

Epileptic seizure could be experienced as traumatic in some patients with PRFE and even induce PTSD. These clinical entities should be explored systematically in clinical practice because its identification and treatment could improve the quality of life of patients with epilepsy. Some therapeutic methods, such as EMDR, cognitive–behavioral therapy, and hypnosis, could be interesting to explore. The neurobiological causes of the links between epilepsy and traumatic events unrelated to epilepsy should also be examined.

## Data Availability Statement

The raw data supporting the conclusions of this article will be made available by the authors, without undue reservation.

## Ethics Statement

The studies involving human participants were reviewed and approved by ethical local comitee. The patients/participants provided their written informed consent to participate in this study.

## Author Contributions

J-PV, CH, LM, and LT conceived of the presented idea and helped supervise the project and designed the study. SM and DV developed the theory and included patients and wrote the manuscript. AT included patients. WE-H and DE wrote the manuscript. JC and SS designed the model and the computational framework and analyzed the data. LM and RS devised the project. CH designed and directed the project. All authors contributed to the article and approved the submitted version.

## Conflict of Interest

The authors declare that the research was conducted in the absence of any commercial or financial relationships that could be construed as a potential conflict of interest.
