# Supplementary material for: Past Trauma Is Associated With a Higher Risk of Experiencing an Epileptic Seizure as Traumatic in Patients With Pharmacoresistant Focal Epilepsy
Source: Front Neurol. 2021 Jul 8;12:669411. doi: 10.3389/fneur.2021.669411 (PMC8296979; doi:10.3389/fneur.2021.669411)
Supplement: Supplementary file 1 [file Table_1.DOCX]

| **Table S1.** Study's population. | |  |
| --- | --- | --- |
| **Demographic data** | |  |
| Age at enrollment, years (SD) | | 33.2 (12.17) |
| Women, n (%) | | 57 (53.28) |
| Living alone, n (%) | | 25 (23.36) |
| Married people, n (%) | | 50 (46.72) |
| Duration of study since first year of primary school, years (SD) | | 12.49 (2.61) |
| Highest diploma validated | |  |
| None, n (%) | | 11 (10.28) |
| 9^th^ grade, n (%) | | 31 (28.97) |
| Vocational certificate obtained 2 years after the 8^th^/9^th^ grade, n (%) | | 11 (10.28) |
| High-school Degree, n (%) | | 27 (25.23) |
| University degree, n (%) | | 27 (25.23) |
| Learning disabilities, n (%) | | 31 (28.97) |
| Student/professional activity, n (%) | | 46 (43) |
| Driving, n (%) | | 32 (29.9) |
| Patient who did not complete at least one questionnaire, n (%) | | 24 (22.42) |
| **Characteristics of epilepsy** | |  |
| Age at the onset of epilepsy, years (SD) | | 17.55 (11.86) |
| Duration of epilepsy, years (SD) | | 15.6 (11.3) |
| MRI lesions, n (%) | | 59 (55.66) |
| **Lateralization** | |  |
| Left-sided epilepsy, n (%) | | 53 (54.63) |
| Right-sided epilepsy, n (%) | | 37 (38.14) |
| Bilateral, n (%) | | 7 (7.21) |
| **Localization** | |  |
| Temporal lobe, n (%) | | 72 (73.46) |
| Frontal lobe, n (%) | | 18 (18.36) |
| Insular cortex, n (%) | | 11 (11,22) |
| Posterior lobe, n (%) | | 14 (14.28) |
| **Type of epilepsy** | |  |
| Focal epilepsy, n (%) | | 95 (88.78) |
| With Focal to bilateral tonic-clonic seizures, n (%) | | 52 (48,59) |
| **Frequencies of seizures** | |  |
| Focal seizures, n per month (SD) | | 27.54 (96.77) |
| Focal to bilateral tonic-clonic seizures, n per month (SD) | | 7.29 (20.6) |
| **Complications during a seizure** | |  |
| Loss of consciousness, n (%) | | 60 (56.60) |
| Urine or feces loss, n (%) | | 21 (19.81) |
| Fall, n (%) | | 43 (40.56) |
| Injuries, n (%) | | 41 (38.67) |
| **Impact of epilepsy on daily life** | |  |
| None, n (%) | | 7 (9.45) |
| Slight, n (%) | | 14 (18.9) |
| Moderate, n (%) | | 29 (39.18) |
| Severe, n (%) | | 14 (18.9) |
| Full, n (%) | | 10 (13.51) |
| **Domains most impacted by epilepsy** | |  |
| None, n (%) | | 6 (8.1) |
| Working life, n (%) | | 54 (72.97) |
| Leisure, n (%) | | 39 (52.7) |
| Sentimental life, n (%) | | 13 (17.56) |
| Family life, n (%) | | 11 (14.86) |
| **Quality of life, QOLIE 31** | |  |
| Overall quality of life, score (SD) | | 63.92 (20.08) |
| Seizure Worry, score (SD) | | 55.23 (27.29) |
| Emotional Well-Being, score (SD) | | 62.94 (22.22) |
| Energy/Fatigue, score (SD) | | 47.41 (18.29) |
| Cognitive, score (SD) | | 57.46 (23.17) |
| Medication Effects, score (SD) | | 61.68 (26.38) |
| Social Function, score (SD) | | 57.46 (25.23) |
| Antiepileptic drugs, n (%) | | 106 (99.06) |
| Average number of AED, n (SD) | | 2.13 (0.84) |
| **Psychiatric assessment, MINI** | |  |
| Number History of psychiatric morbidities, n (SD) | | 1.19 (1.14) |
| Number Current psychiatric comorbidities, mean (SD) | | 0.85 (0.88) |
| At least one psychiatric history, n (%) | | 72 (67.28) |
| At least one current pathology, n (%) | | 64 (59.81) |
| **Current psychotropic treatment** |  | |
| Anti-depressant, n (%) | | 11 (10.28) |
| Neuroleptic, n (%) | | 7 (6.54) |
| Anxiolytic, n (%) | | 8 (7.47) |
| Number of psychotropic drugs, n (SD) | | 0.24 (0.63) |
| **Personal psychiatric history** |  | |
| Psychiatric follow-up, n (%) | | 48 (44.85) |
| Hospitalization for psychiatric reason, n (%) | | 13 (12.14) |
| Personal history of suicide attempt, n (%) | | 12 (11.21) |
| Family psychiatric history, n (%) | | 28 (26.16) |
| **Mood disorders** | | **18 (16.82)** |
| Positive NDDIE (score ⩾ 16), n (%) | | 18 (18.55) |
| Depressive disorder (current), n (%) | | 12 (11.21) |
| Hypomania/Mania (current), n (%) | | 0 (0) |
| Dysthymia (current), n (%) | | 10 (9.34) |
| Bipolar disorder (current), n (%) | | 0 (0) |
| Interictal dysphoric disorder, n (%) | | 25 (23.36) |
| Pre-ictal mood disorder, n (%) | | 8 (7.47) |
| Ictal mood disorder, n (%) | | 3 (2.8) |
| Post-ictal mood disorder, n (%) | | 16 (14.95) |
| Mood disorder induced by an anti-epileptic treatment, n (%) | | 27 (25.23) |
| **Anxious disorders** | | **34 (31.77)** |
| Positive GAD 7 (score ⩾ 8), n (%) | | 26 (26.8) |
| Panic disorder (current), n (%) | | 8 (7.47) |
| Agoraphobia (current), n (%) | | 9 (8.41) |
| Generalized anxiety disorder (current), n (%) | | 13 (12.14) |
| Social phobia (current), n (%) | | 8 (7.47) |
| Obsessive-compulsive disorder (current), n (%) | | 4 (3.73) |
| PTSD (current; not related to epilepsy), n (%) | | 5 (4.67) |
| PTSD (past ; not related to epilepsy), n (%) | | 14 (13.08) |
| Pre-ictal anxiety, n (%) | | 35 (32.71) |
| Interictal anxiety | |  |
| Anticipatory anxiety of a seizure, n (%) | | 38 (52.05) |
| Ictal anxiety, n (%) | | 35 (32.71) |
| Post-ictal anxiety, n (%) | | 15 (14.01) |
| Behaviors of limitation or avoidance, n (%) | | 44 (41.12) |
| Anxious disorder induced by an anti-epileptic treatment, n (%) | | 7 (6.54) |
| Eating disorders, n (%) | | 7 (6.54) |
| Eating disorder induced by an anti-epileptic treatment, n (%) | | 29 (27.1) |
| **Addictive disorders** | | **28 (26.16)** |
| Tobacco addiction (current), n (%) | | 26 (24.29) |
| Alcohol addiction (current), n (%) | | 3 (2.8) |
| Cannabis addiction (current), n (%) | | 5 (4.67) |
| Other drug addiction (current), n (%) | | 1 (0.93) |
| Gambling addiction (current), n (%) | | 1 (0.93) |
| Benzodiazepine addiction (current), n (%) | | 0 (0) |
| **Psychotic disorders** | | **10 (9.34)** |
| Psychotic disorder (current), n (%) | | 1 (0.93) |
| Post-ictal psychosis, n (%) | | 1 (0.93) |
| Psychotic symptomatology induced by an anti-epileptic treatment, n (%) | | 7 (6.54) |
| **Alexithymia, TAS** | |  |
| Alexithymia (score > 61), n (%) | | 18 (20.45) |
| Feelings' description difficulties, score (SD) | | 13.14 (3.97) |
| Feelings' identification difficulties, score (SD) | | 17.64 (6.27) |
| Thoughts turned to the outside, score (SD) | | 20.59 (4.07) |
| **Dissociation severity, DES** | |  |
| Total score, (SD) | | 9.88 (8.43) |
| Depersonalization, score (SD) | | 10.04 (8.99) |
| Amnesia, score (SD) | | 6.83 (7.19) |
| Absorption, score (SD) | | 14.81 (13.56) |

*SD: Standard Deviation;* *QOLIE: Quality Of Life in Epilepsy Inventory, MINI: Mini International Neuropsychiatric Interview; NDDI-E: Neurological Disorders Depression Inventory for Epilepsy; GAD: Generalized Anxiety Disorder; PS-PTSD: Post Seizure Post Traumatic Stress Disorder; TAS: Toronto Alexithymia Scale; CTQ: Childhood Trauma Questionnaire; PCL-5: Post-Traumatic Stress Disorder Checklist for DSM-5; DES: Dissociative Experiences Scale*
